# Patient Portal Functionalities and Patient Outcomes Among Patients With Diabetes: Systematic Review

**DOI:** 10.2196/18976

**Published:** 2020-09-22

**Authors:** Abrar Alturkistani, Ambar Qavi, Philip Emeka Anyanwu, Geva Greenfield, Felix Greaves, Ceire Costelloe

**Affiliations:** 1 Global Digital Health Unit Department of Primary Care and Public Health Imperial College London London United Kingdom; 2 Department of Primary Care and Public Health Imperial College London London United Kingdom; 3 Public Health Policy Evaluation Unit Department of Primary Care and Public Health Imperial College London London United Kingdom

**Keywords:** personal health record, patient portal, electronic health records, online access, patient records, systematic review

## Abstract

**Background:**

Patient portal use could help improve the care and health outcomes of patients with diabetes owing to functionalities, such as appointment booking, electronic messaging (e-messaging), and repeat prescription ordering, which enable patient-centered care and improve patient self-management of the disease.

**Objective:**

This review aimed to summarize the evidence regarding patient portal use (portals that are connected to electronic health care records) or patient portal functionality use (eg, appointment booking and e-messaging) and their reported associations with health and health care quality outcomes among adult patients with diabetes.

**Methods:**

We searched the MEDLINE, Embase, and Scopus databases and reported the review methodology using the Preferred Reporting Items for Systematic Reviews and Meta-Analyses (PRISMA) guidelines. Three independent reviewers screened titles and abstracts, and two reviewers assessed the full texts of relevant studies and performed data extraction and quality assessments of the included studies. We used the Cochrane Collaboration Risk of Bias Tool and the National Heart, Lung and Blood Institute (NHLBI) Study Quality Assessment Tool to assess the risk of bias of the included studies. Data were summarized through narrative synthesis.

**Results:**

Twelve studies were included in this review. Five studies reported overall patient portal use and its association with diabetes health and health care quality outcomes. Six studies reported e-messaging or email use–associated outcomes, and two studies reported prescription refill–associated outcomes. The reported health outcomes included the associations of patient portal use with blood pressure, low-density lipoprotein cholesterol, and BMI. Few studies reported health care utilization outcomes such as office visits, emergency department visits, and hospitalizations. A limited number of studies reported overall quality of care for patients with diabetes who used patient portals.

**Conclusions:**

The included studies mostly reported improved glycemic control outcomes for patients with diabetes who used patient portals. However, limitations of studying the effects of patient portals exist, which do not guarantee whether the outcomes reported are completely the result of patient portal use or if confounding factors exist. Randomized controlled trials and mixed-methods studies could help understand the mechanisms involved in health outcome improvements and patient portal use among patients with diabetes.

**Trial Registration:**

International Prospective Register of Systematic Reviews (PROSPERO) CRD42019141131; https://www.crd.york.ac.uk/prospero/display_record.php?ID=CRD42019141131.

**International Registered Report Identifier (IRRID):**

RR2-10.2196/14975

## Introduction

### Background

Patient portals are online tools connected to health care systems’ electronic health records (EHRs). The portals may improve patient health outcomes by improving communication with health care providers, enabling self-management of the disease, increasing patients’ involvement in care, empowering patients, and improving their knowledge about the disease [1-6]. By offering access to information, such as visit summaries and health records, portals can help patients review information and remember doctors’ instructions [7,8]. Asynchronous communication with health care providers through electronic messages (e-messages) or emails (a potential functionality of patient portals) can increase patients’ interaction with the health care system and enable continuity of care [3,5,9,10]. Services, such as repeat prescription refills through the portal, could also improve efficiency and accelerate medication dispensing [3].

### Rationale

The World Health Organization recommends a patient-centered approach when it comes to diabetes care and the use of technologies to engage patients [11]. The chronic care model, an evidence-based approach to manage chronic diseases, recommends “self-management support” to provide the best care for patients with chronic diseases [12]. Synthesizing and weighting the evidence about patient portals’ effectiveness in improving diabetes health outcomes and quality of care could help inform health care professionals and policymakers about the potential benefits of patient portals.

There are several systematic reviews about patient portals used by patients with diabetes. However, published reviews are either outdated owing to new studies about patient portals being published in the last 2 to 3 years [13-16] or do not report patient outcomes [13]. Previous reviews that looked at diabetes health outcomes associated with portal use had a broader definition of portals and included portals that are co-delivered with other interventions, such as a diabetes management system, home visits [14], and coaching programs [16]. Another review only reported on the user characteristics of patients with diabetes, as well as facilitators and barriers of portal use [13], but did not report on the health outcomes associated with portal use. We hence aimed to close this gap by conducting a systematic review to summarize and evaluate the study findings that reported health and health care quality outcomes associated with the use of patient portals among adult patients with diabetes.

### Objective

We aimed to summarize the evidence regarding the use of patient portals (portals that are connected to EHRs) and its reported association with health and health care quality outcomes among adult patients with diabetes. The review research questions were as follows: (1) What kind of health outcomes do patient portals contribute to in adult patients (18 years or older) with diabetes? (2) What kind of health care quality outcomes, including health care utilization outcomes, do patient portals contribute to in adult patients (18 years or older) with diabetes?

## Methods

### Guidelines and Study Registration

This review was conducted following the Preferred Reporting Items for Systematic Reviews and Meta-Analyses (PRISMA) guidelines [17] (Multimedia Appendix 1). The protocol of the review was registered in the International Prospective Register of Systematic Reviews (PROSPERO) (registration number: CRD42019141131) and was published in JMIR Research Protocols (RR1-10.2196/14975) [18]. 

### Eligibility Criteria

The population included adult patients with diabetes aged 18 years or older. The initial intent in the review protocol [18] was to include all adult patient portal users, without focusing on patients with a specific disease. However, owing to the large number of studies reporting patient portal–related health outcomes and the diversity of the patient populations studied, we decided to focus on patients with diabetes only. The intervention only included tethered patient portals that are connected to a health care system’s EHR. We excluded studies with additional interventions besides the patient portal, such as a wearable device and a portal with a mood monitoring tool [19], as we were unable to determine the outcomes associated with portal use only. Studies with comparators and no comparators were included. Outcomes of interest were health or health care quality outcomes. Qualitative and conference papers were excluded. Mixed-methods studies were only included if the quantitative results were of the outcomes of interest of the study. Finally, we excluded usability-only studies.

### Information Sources

The MEDLINE, Embase, and Scopus databases were searched for relevant articles. The complete search strategy for each database has been provided in Multimedia Appendix 2. The search was performed up to September 2019, but there was no restriction on the start date of the search. 

### Study Selection and Data Extraction

Three reviewers independently performed title and abstract screening. Two reviewers (AA and AQ) independently assessed all full texts for eligibility, while a third reviewer (PEA) performed 25% (5 out of 20 articles) of the full-text reading. Data extraction was also performed independently by the two reviewers (AA and AQ). The extracted data included study design, population characteristics, patient portal characteristics, and study outcomes, and extraction was performed using the Cochrane primary screening and data extraction tool (Covidence) [20]. Any conflicts between the two reviewers were resolved through discussions with the third reviewer (PEA). 

### Risk of Bias

Studies were assessed for risk of bias using the Cochrane Collaboration Risk of Bias Tool [21] for randomized controlled trials (RCTs) and the National Heart, Lung and Blood Institute (NHLBI) Study Quality Assessment Tool for observational cohort and cross-sectional studies [22]. The NHLBI tool helps identify the “internal validity of studies” by guiding the users to identify methodological limitations [22]. Although studies are rated as good, fair, or poor, the tool does not assign numeric values or definite judgements of the quality of the studies. Thus, it is up to the authors’ judgement to decide the severity of the risk of bias in studies using the guidance questions. In this review, we considered “good” as having a low risk of bias, “fair” as having a moderate risk of bias, and “poor” as having a high risk of bias. The risk of bias outcomes were considered when interpreting study findings in the discussion section.

### Data Synthesis

We were unable to carry out a meta-analysis owing to the variation in outcomes and methodologies used in the included studies. Therefore, we conducted a narrative synthesis of the results from the included studies based on the study designs and outcomes reported, paying attention to the relationship between the studies. We examined the relationship between the studies based on the patient portal functionalities. We decided to only report the outcomes of interest in relation to the patient portal functionality use because we found that there were similar patterns in the outcomes reported based on the functionalities. Although we collected information about the health care setting of each of the included studies, we did not find sufficient information to judge if the health care setting had a relevant influence on the outcomes. Thus, we did not compare study findings based on the health care setting. The narrative synthesis followed the methodologies proposed by the Cochrane Consumers and Communication Review Group data synthesis and analysis document [23] and the methodologies proposed as part of the UK Economic and Social Research Council Methods Program [24].

## Results

### Study Selection

**Figure 1:**
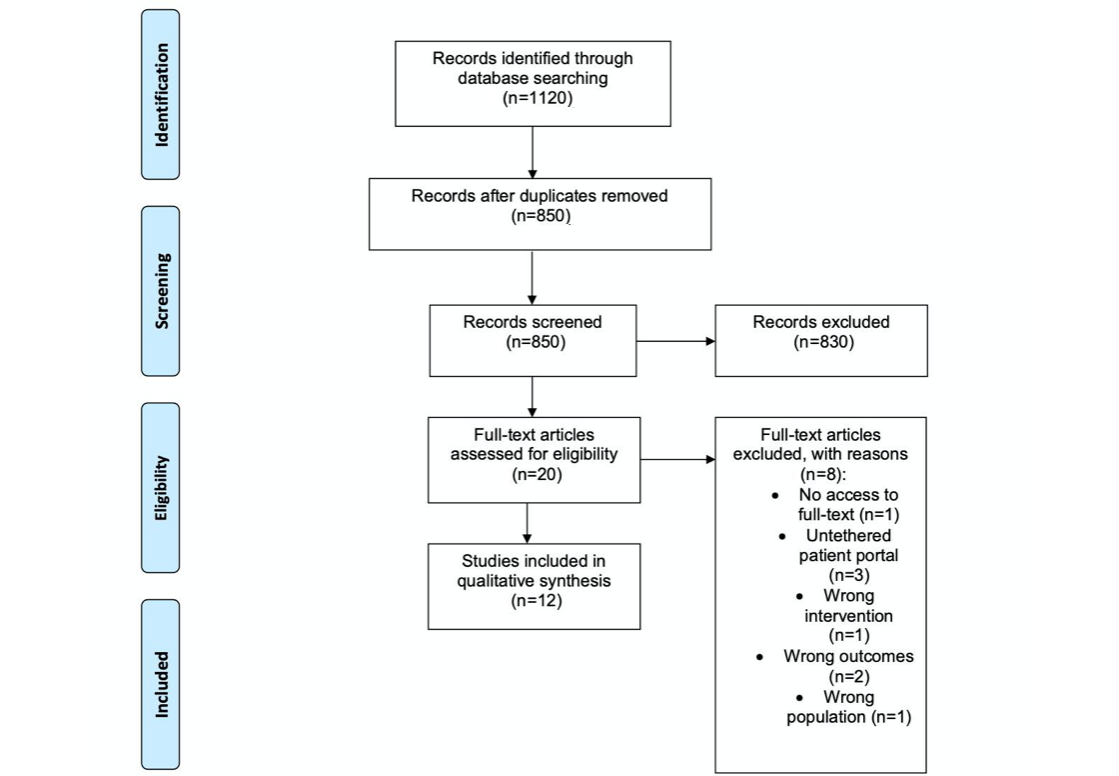
Preferred Reporting Items for Systematic Reviews and Meta-Analyses (PRISMA) flow diagram of the search and study selection process.

A total of 1120 records were initially identified from the database searches (Figure 1). Among these, 830 studies were excluded after title and abstract screening (conference papers and irrelevant studies were excluded at this stage). Twenty full texts were assessed for eligibility, of which eight were excluded (Multimedia Appendix 3). Eventually, 12 studies were included in the final review, and a narrative synthesis was performed (Figure 1).

### Characteristics of the Included Studies

A summary table of the characteristics of the included studies is provided in Multimedia Appendix 4. Most of the studies were from the United States (n=11), with only one study from Canada [25]. Studies were performed in mixed settings including primary, secondary, and tertiary care (outpatient setting), and about half of the studies only included patients with type 2 diabetes. The primary care setting included patient portals offered by patients’ primary care providers. The secondary care setting included patient portals provided in a hospital setting. The tertiary care setting included specialized outpatient care (eg, diabetologists). Integrated health care systems included primary care services provided in a hospital setting. None of the studies specified whether the patient portal was used in an in-patient setting. The retrospective cohort study design was the design of choice in most cases, and there was one RCT [26] and one cross-sectional study [27].

Table 1 lists the different patient portal functionalities used in the included studies. The functionalities varied between studies but mostly included viewing laboratory results (n=9), scheduling appointments (n=6), refilling medications (n=7), and sending messages or emails to health care providers (n=12) [25-36]. Other functionalities of the portals included requesting medical advice, updating demographic information by patients [28], entering flowsheet data, and offering a patient journal [25]. The intervention portal in the RCT [26] allowed patients to edit their medication lists, collected patient data on adverse effects of medication and response to therapy, and allowed patients to raise their health concerns through the portal. The control arm (patient portal) of the RCT included similar functionalities to the intervention portal and allowed patients to enter family medical history and review their patient records concerning nondiabetes-related health concerns such as cancer screening [26].

**Table 1:** Patient portal functionalities in the included studies.

Study		Patient portal functionalities						
		Lab results	Visit notes	Appointment booking	Repeat medication refill	Secure message/email	Patient education	Other
**Randomized controlled trials**								
	Grant et al, 2008 [26]	Yes^a^	No^b^	Yes	Yes	Yes	No	Yes
**Retrospective cohort studies**								
	Chung et al, 2017 [28]	Yes	Yes	Yes	Yes	Yes	Yes	Yes
	Devkota et al, 2016 [29]	Yes	No	No	No	Yes	Yes	Yes
	Lau et al, 2014 [25]	Yes	No	No	No	Yes	Yes	Yes
	Lyles et al, 2016 [30]	Yes	Yes	Yes	Yes	Yes	No	No
	McClellan et al, 2016 [31]	Yes	No	No	Yes	Yes	No	Yes
	Petullo et al, 2016 [32]	No	No	No	No	Yes	No	Yes
	Price-Haywood et al, 2017 [33]	No	No	Yes	Yes	Yes	No	Yes
	Reed et al, 2019 [34]	Yes	Yes	Yes	Yes	Yes	No	No
	Shimada et al, 2016 [35]	No	No	No	Yes	Yes	No	Yes
	Tenforde et al, 2012 [36]	Yes	No	No	No	Yes	Yes	No
**Cross-sectional studies**								
	Wade-Vuturo et al, 2013 [27]	Yes	No	Yes	No	Yes	No	Yes

^a^Yes indicates availability of the functionality.

^b^No indicates nonavailability or no mention of the functionality in the study.

### Risk of Bias

The RCT included in this review [26] had a slightly high risk of bias (Multimedia Appendix 5) stemming from not being able to blind study participants or outcome assessors to the exposure status of participants. The RCT also had a high risk of bias owing to not reporting some of the study outcomes despite mentioning the outcomes in the methods section [26].

We rated most of the observational studies as having a low or moderate risk of bias (Multimedia Appendix 6). The studies that we rated as having a low or moderate risk of bias generally measured exposure before the outcomes [29,30,32-36], measured different levels of exposure (eg, compared portal functionality use by the number of days or number of times the functionality was used instead of having only one category for usage) [29-32,35,36], and controlled for key confounding variables [29-31,34-36]. Few studies that looked at the frequency or volume of e-messages, emails, or prescription refill use found that patients who used the functionality the most had better outcomes than patients who did not use the functionality or who used it less frequently. For example, one study reported that only patients who both read and wrote emails had much better glycemic control at follow up (odds ratio [OR] 1.43, 95% CI 1.11-1.83), which was not true for patients only reading emails or only using the patient portal [29]. Similarly, another study found that the odds of glycemic control was the highest among patients using the e-messaging functionality for 3 years or more (OR 1.28, 95% CI 1.13-1.44) compared with portal-only users (using the patient portal without the e-messaging functionality) [35]. One study found that patients sending four or more messages per year were more likely to meet the glycemic control threshold compared with those sending one message only (OR 1.55, 95% CI 1.43-1.69) [28].

### Health Outcomes

Table 2 summarizes the different outcomes reported to be associated with patient portal use, e-messaging or emailing, and medication refill through the portal. Overall, patient portal use was reported to be associated with glycemic control, reduced glycated hemoglobin A_1c_ (HbA_1c_%) at follow up, reduced blood pressure, increased office visits, reduced hospitalizations, medication adherence, and medication adjustment. One study did not find a significant improvement in glycemic control as a result of using a patient portal (*P*=.62); however, the study offered both the control and experimental groups access to a patient portal [26]. E-message or email use was reported to be associated with glycemic control, reduced HbA_1c_% at follow up, reduced low-density lipoprotein cholesterol, and increased office visits. Only one study examined the difference in BMI among portal users and nonusers and found no relevant difference [36]. Refilling medications through the patient portal was reported to be associated with glycemic control, blood pressure control, and medication adherence [35]. Only one study reported an association between refilling medications exclusively through the patient portal and improved statin adherence [30]*.* Although not listed in Table 2, one study found no correlation regarding reviewing laboratory results; viewing medical records; accessing billing information, the telephone directory, maps/directions, and insurance information; finding a doctor; and paying medical bills through the portal [27].

**Table 2:** Patient portal or patient portal functionality use and the reported associations with diabetes health and health care outcomes in the included studies.

Outcome	Overall patient portal use (n=5)	Electronic messaging or email use (n=6)	Prescription refill use (n=2)
Glycemic control	Positive association (n=1) [25], no association (n=1) [26]	Positive association (n=3) [28,29,35], weak correlation (n=1) [27], no association (n=1) [31]	Positive association (n=1) [35]
Hemoglobin A_1c_ (HbA_1c_%) at follow up	Inverse association (n=3) [25,33,36]	Inverse association (n=3) [29,31,32]	—^a^
Low-density lipoprotein cholesterol	No association (n=2) [25,36]	Inverse association (n=1) [35], no association (n=1) [31]	Inverse association (n=1) [35]
Blood pressure	Inverse association (n=1) [36], no association (n=1) [25]	No association (n=2) [31,35]	Inverse association (n=1) [35]
BMI	No association (n=1) [36]	—	—
Office visits	Positive association (n=1) [34]	Positive association (n=1) [28]	—
Emergency visits	Inverse association (n=1) [34]	No association (n=1) [32]	—
Hospitalization	Inverse association (n=1) [34]	No association (n=1) [32]	—
Medication adherence	—	—	Positive association (n=1) [30]
Medication adjustment	Positive association (n=1) [26]	—	—

^a^There were no studies reporting an association between functionality and outcome.

### Diabetes Care Quality Outcomes

Three studies reported that patient portal or e-message users were more likely to meet most of the diabetes care standards, such as the Diabetes Healthcare Effectiveness Data and Information Set (HEDIS) quality measures [28,31], or the diabetes standards by the Better Health Partnership: Diabetes Standards [36]. 

## Discussion

### Review of the Findings

Our review found a limited number of studies examining the association between patient portal use and diabetes health and health care quality outcomes. Nevertheless, among the studies included, patient portal use or patient portal functionality use was reported to be associated with improvements in health outcomes, such as glycemic control. Secure messaging or emailing, or repeat prescription ordering through the patient portal was reported to be associated with improved glycemic control, and outcomes appeared to improve with increased use. It was also reported that patient portal use may be associated with improved low-density lipoprotein cholesterol outcomes or blood pressure control. Patient portal use or patient portal functionality use might affect health care utilization and may be associated with increased office visits and decreased emergency department visits. Finally, some of the included studies suggested that patient portal use might be associated with improved quality of care for patients with diabetes.

The majority of studies we reviewed were determined to have low to moderate risk of bias. However, some factors may not be measured through standard risk of bias tools, which might have affected the results reported by the studies. For instance, it is challenging to separate outcomes that result exclusively from portal use owing to the possibility of the presence of other factors that might confound the association. It is also challenging to conclude which functionality of the patient portal contributes the most to improving health outcomes, as some studies only report overall portal use. It was previously reported that secure messaging improves health outcomes for patients with diabetes [14]. Increased contact between patients with diabetes and health care professionals was one of the functionalities most associated with reduced HbA_1c_ in diabetes disease-management programs as reported in a meta-analysis of studies [37]. Our review also included studies that suggest glycemic control is improved in patients who use secure messaging owing to improved communication and increased access to care [29], resulting in “better diabetes management” [28].

Outcomes related to the quality of care and health care utilization were mixed. While some studies found reductions in emergency visits, others did not. A previous study in a diverse patient population found that there was no association between patient portal use, hospital admission, and 30-day readmission, suggesting that patient portals could be more effective in managing chronic care than acute care [38]. Alternatively, one of the included studies in this review suggested that actions related to portal use, such as checking a test result, can increase office visits, while actions, such as repeat prescription ordering during the after-hours period, might reduce hospitalizations [34]. A recent survey study of a patient portal with access to health care records, test results, e-messaging, and appointment booking reported that patients believed portal use helped them “avoid a clinic visit” [39]. Few studies in the literature also examined the association between patient portal use and missing medical appointments [39,40], which was not examined in any of the studies included in this review. The health care utilization outcomes associated with patient portal use may need further investigation as the number of studies examining these associations is limited.

### Knowledge Gap

The outcomes of this review indicate that there remain persistent gaps in the literature about patient portals used by patients with diabetes. First, there is some evidence that increased frequency of patient portal or patient portal functionality use could be associated with increased benefits, suggesting a dose-response relationship. Patient portal adoption does not indicate continuous use [40]. Since differences in the frequency of use may lead to inconsistencies in benefits acquired from the patient portal, studies need to account for the frequency of patient portal use as much as possible. Additionally, there is a need for mixed-methods studies to evaluate the mechanisms through which portal use might impact the outcomes reported. Further examination of health care utilization outcomes could help understand if patient portals can play a role in improving health care utilization patterns among patients with diabetes.

### Limitations

The results reported by the studies in this review could be biased owing to factors that may not have been controlled. For example, patient portal users can be generally more motivated to be involved in their care and to improve their health outcomes [29]. A cross-sectional study reported that patients who preferred using portals had higher “self-determination” to manage their health conditions [41]. RCTs could help explore causal relationships between portal use and outcomes in patients with diabetes. Additionally, qualitative studies or mixed-methods studies can help explain if portal use or patient portal functionality use is responsible for improving health and quality of care outcomes among patients with diabetes. Qualitative studies could help explore patients’ motives and patterns in self-management to further help understand the mechanisms involved in improving health outcomes through patient portal use. There continues to be a need for studies to report outcomes based on functionality whenever possible [16].

Although this review tried to report portal functionality–related outcomes along with overall portal-related outcomes, most included studies did not sufficiently report outcomes by functionality. Another limitation of this review is that all studies, except one, were from the United States, which has a diverse health care system involving private health care organizations, nonprofit organizations, and government-owned organizations. The way that the health care organization is organized may limit the application of the findings of this review to other health care settings and systems.

Another limitation of this review is the small number of studies included. The limited number of studies reduced the generalizability of the review findings. However, the review only attempted to identify associations as reported by the included studies, which warrants further appropriately designed studies in order to assess causal associations.

### Conclusion

Most of the included studies reported improved glycemic control outcomes for patients with diabetes who used patient portals. However, limitations of studying the effects of patient portals exist, which do not guarantee whether the outcomes reported are completely the result of patient portal use or if confounding factors exist. RCTs and mixed-methods studies could help understand the mechanisms involved in health outcome improvements and patient portal use among patients with diabetes. 

## Appendix

Multimedia Appendix 1 PRISMA 2009 checklist.

Multimedia Appendix 2 Search strategy.

Multimedia Appendix 3 List of excluded studies with reasons for exclusion.

Multimedia Appendix 4 Characteristics of the included studies.

Multimedia Appendix 5 Risk of bias assessment results on applying the Cochrane Collaboration Risk of Bias Tool for randomized controlled trials by Grant et al [26].

Multimedia Appendix 6 Risk of bias assessment results on applying the National Heart, Lung and Blood Institute quality assessment tool for observational cohort and cross-sectional studies.

## Abbreviations

EHR: electronic health record
OR: odds ratio
RCT: randomized controlled trial
