# Supplementary material for: Patient Portal Functionalities and Patient Outcomes Among Patients With Diabetes: Systematic Review
Source: J Med Internet Res. 2020 Sep 22;22(9):e18976. doi: 10.2196/18976 (PMC7539164; doi:10.2196/18976)
Supplement: Multimedia Appendix 2 [file jmir_v22i9e18976_app2.docx]

**Multimedia Appendix 2: [Search strategy]**

**MEDLINE search through OVID search strategy:**

Patient Portals/

Patient* adj3 portal*

electronic personal health record*

health* portal*

online portal*

personal health record* (title only)

personal medical record*

patient accessible electronic health record*

patient accessible electronic medical record*

personal health information management system*

Patient platform*

((Patient Access to Records/ OR patient* adj3 access* OR Personal) AND (Electronic Health Records/ OR electronic medical record*)) (title only)

Web portal* AND (patient* or personal)

((electronic portal*) AND (patient* or personal) ) Not (Imaging)

(Web-based adj1 portal*) AND (patient* or personal)

1 OR 2 OR 3 OR 4 OR 5 OR 6 OR 7 OR 8 OR 9 OR 10 OR 11 OR 12 OR 13 OR 14 OR 15

exp Diabetes Mellitus/

insulin* depend* or non insulin* depend*

IDDM or NIDDM or MODY or T1DM or T2DM or T1D or T2D

diabet*

17 OR 18 OR 19 OR 20

16 AND 21

Total results: 197

(No limits)

*An asterisk helps find the variations of the word with the same stem. For example, portal* will detect both the words portal and portals.

**EMBASE through OVID search strategy:**

(Patient adj3 portal*) NOT (vein OR “portal hypertension” or liver)

electronic personal health record*

health* portal*

online portal*

personal health record* (title only)

personal medical record*

patient accessible electronic health record*

patient accessible electronic medical record*

personal health information management system*

Patient platform*

((Patient Access to Records/ OR patient* adj3 access* OR Personal) AND (Electronic Health Records/ OR electronic medical record*)) (title only)

Web portal* AND (patient* or personal)

((electronic portal*) AND (patient* or personal) ) Not (Imaging)

(Web-based adj1 portal*) AND (patient* or personal)

1 OR 2 OR 3 OR 4 OR 5 OR 6 OR 7 OR 8 OR 9 OR 10 OR 11 OR 12 OR 13 OR 14

exp diabetes mellitus/

non insulin dependent diabetes mellitus/ or insulin dependent diabetes mellitus/

diabet*

non insulin* depend* or insulin* depend*

IDDM or NIDDM or MODY or T1DM or T2DM or T1D or T2D

16 OR 17 OR 18 OR 19 OR 20 OR 21

15 AND 21

Total results: 294

(No limits)

*An asterisk helps find the variations of the word with the same stem. For example, portal* will detect both the words portal and portals.

**Scopus search strategy:**

“electronic personal health record*”

“health* portal*”

“Online patient portal*”

“personal health record*” (title only)

“personal medical record*”

“patient accessible electronic health record*”

“patient accessible electronic medical record*”

“personal health information management system*”

“Patient platform*”

(“patient* access*” OR “Personal”) AND (“Electronic Health* Record*” OR “electronic medical record*”)) (title only)

“Web portal*” AND (patient* or personal)

(“electronic portal*” AND (patient* or personal) ) AND NOT (Imaging)

(“Patient* w/3 portal*” AND NOT (vein) OR (liver) OR “portal hypertension”)

(Web-based w/2 portal*) AND (patient* or personal)

1 OR 2 OR 3 OR 4 OR 5 OR 6 OR 7 OR 8 OR 9 OR 10 OR 11 OR 12 OR 13 OR 14

diabet*  OR  iddm  OR  niddm  OR  mody  OR  t1dm  OR  t2dm  OR  t1d  OR  t2d  OR  ( insulin*  w/2  depend* )

15 and 16

(Excluded conference papers and reviews)

 Total results: 629

*An asterisk helps find the variations of the word with the same stem. For example, portal* will detect both the words portal and portals.

w/2 and w/3 are the within operators in Scopus and they help find literature that uses the searched words within 2 or 3 words to each other. For example, patient w/3 portal will detect  all of the following: patient portal, patient web portal and patient electronic web portal.
