# Supplementary material for: Patient Portal Functionalities and Patient Outcomes Among Patients With Diabetes: Systematic Review
Source: J Med Internet Res. 2020 Sep 22;22(9):e18976. doi: 10.2196/18976 (PMC7539164; doi:10.2196/18976)
Supplement: Multimedia Appendix 3 [file jmir_v22i9e18976_app3.docx]

**Multimedia Appendix 3: [List of excluded studies with the reason of exclusion]**

| Study title and reference | Reason for exclusion |
| --- | --- |
| Early Adopters of Patient-Generated Health Data Upload in an Electronic Patient Portal [1] | No access to full-text (Publisher under one-year embargo in UK libraries) |
| Evaluating the Effect of Web-Based Iranian Diabetic Personal Health Record App on Self-Care Status and Clinical Indicators: Randomized Controlled Trial [2] | Untethered patient portal |
| Performance of an Electronic Diary System for Intensive Insulin Management in Global Diabetes Clinical Trials [3] | Wrong intervention |
| Value versus user fees: Perspectives of patients before and after using a web-based portal for management of diabetes [4] | Not reporting outcomes of interest |
| Users' Attitudes Towards Personal Health Records: A Cross-Sectional Pilot Study [5] | Untethered patient portal |
| Mobile Diabetes Intervention Study of Patient Engagement and Impact on Blood Glucose: Mixed Methods Analysis [6] | Untethered patient portal |
| Patient-provider internet portals--patient outcomes and use [7] | Wrong patient population- study included patients younger than 18 year old |
| Patients' Experiences with and Attitudes towards a Diabetes Patient Web Portal [8] | Not reporting outcomes of interest |

# REFERENCES

1. Ancker, Jessica S., Elizabeth Mauer, Robin B. Kalish, Joshua R. Vest, and J. Travis Gossey. ‘Early Adopters of Patient-Generated Health Data Upload in an Electronic Patient Portal’. *Applied Clinical Informatics* 10, no. 2 (March 2019): 254–60. <https://doi.org/10.1055/s-0039-1683987>.
2. Azizi, Amirabbas, Robab Aboutorabi, Zahra Mazloum-Khorasani, Monavar Afzal-Aghaea, Hamed Tabesh, and Mahmood Tara. ‘Evaluating the Effect of Web-Based Iranian Diabetic Personal Health Record App on Self-Care Status and Clinical Indicators: Randomized Controlled Trial’. *JMIR Medical Informatics* 4, no. 4 (October 2016): e32.
3. Bastyr, Edward J. 3rd, Shuyu Zhang, Jiani Mou, Andy P. Hackett, Stephen A. Raymond, and Annette M. Chang. ‘Performance of an Electronic Diary System for Intensive Insulin Management in Global Diabetes Clinical Trials’. *Diabetes Technology & Therapeutics* 17, no. 8 (August 2015): 571–79. <https://doi.org/10.1089/dia.2014.0407>.
4. Bryce, Cindy L., Susan Zickmund, Rachel Hess, Kathleen M. McTigue, Ellen Olshansky, Katharine Fitzgerald, and Gary Fischer. ‘Value versus User Fees: Perspectives of Patients before and after Using a Web-Based Portal for Management of Diabetes’. *Telemedicine Journal and E-Health: The Official Journal of the American Telemedicine Association* 14, no. 10 (December 2008): 1035–43. <https://doi.org/10.1089/tmj.2008.0005>.
5. Khaneghah, Peyman Azad, Antonio Miguel-Cruz, Pamela Bentley, Lili Liu, Eleni Stroulia, and Martin Ferguson-Pell. ‘Users’ Attitudes Towards Personal Health Records: A Cross-Sectional Pilot Study’. *Applied Clinical Informatics* 7, no. 2 (2016): 573–86. <https://doi.org/10.4338/ACI-2015-12-RA-0180>.
6. Quinn, Charlene C., Ann L. Gruber-Baldini, Michelle Shardell, Kelly Weed, Suzanne S. Clough, Malinda Peeples, Michael Terrin, Lauren Bronich-Hall, Erik Barr, and Dan Lender. ‘Mobile Diabetes Intervention Study: Testing a Personalized Treatment/Behavioral Communication Intervention for Blood Glucose Control’. *Contemporary Clinical Trials* 30, no. 4 (July 2009): 334–46. <https://doi.org/10.1016/j.cct.2009.02.004>.
7. Ronda, Maaike C. M., Lioe-Ting Dijkhorst-Oei, and Guy E. H. M. Rutten. ‘Patients’ Experiences with and Attitudes towards a Diabetes Patient Web Portal’. *PloS One* 10, no. 6 (2015): e0129403. <https://doi.org/10.1371/journal.pone.0129403>.
8. Shaw, Ryan J., and Jeffrey Ferranti. ‘Patient-Provider Internet Portals--Patient Outcomes and Use’. *Computers, Informatics, Nursing: CIN* 29, no. 12 (December 2011): 714–18; quiz 719–20. <https://doi.org/10.1097/NCN.0b013e318224b597>.
