# Supplementary material for: Patient Portal Functionalities and Patient Outcomes Among Patients With Diabetes: Systematic Review
Source: J Med Internet Res. 2020 Sep 22;22(9):e18976. doi: 10.2196/18976 (PMC7539164; doi:10.2196/18976)
Supplement: Multimedia Appendix 4 [file jmir_v22i9e18976_app4.docx]

**Multimedia Appendix 4: [Characteristics of included studies]**

| **Author(s)** | **Country** | **Setting** | **Participants** | **Sample size** | **Type of diabetes** |
| --- | --- | --- | --- | --- | --- |
| 1. Randomised clinical trial | | | | | |
| Grant et al., 2008 [26] | USA | Primary care | Patients with HbA1c level > 7.0% in the previous year, with active diabetes medication and one visit to the primary care physician in the previous year, with an active patient portal account. | 244 | Type 2 |
| 2. Retrospective cohort studies | | | | | |
| Chung et al., 2017 [28] | USA | Primary care | Primary care patients aged 18-75 with at least 1 visit to primary care during the year, enrolled in the patient portal. | 20,655 | Type 2 |
| Devkota et al., 2016 [29] | USA | Primary care | Patients with at least one visit and a documented HbA1c value in observation period | 1,510 | Type 2 |
| Lau et al., 2014 [25] | Canada | Tertiary care (Diabetologist) | Patients 18 years or older, with recorded demographic data, initial A1c score, and a follow up A1c score between 6-24 months later. | 157 | Unspecified |
| Lyles et al., 2016 [30] | USA | Integrated healthcare system | Portal user prescribed statins for the entire study period and refilling statin prescriptions exclusively within the healthcare system. | 17,760 | Type 2 |
| McClellan et al., 2016 [31] | USA | Integrated healthcare system | Patients 18 years or older, with at least 1 visit/24 months, who have a designated primary care provider within the study location, had at least one charge and diagnosed with diabetes or hypertension. | 4,232 | Unspecified |
| Petullo et al., 2016 [32] | USA | Secondary care | Patients with at least 1 visit to the endocrinology clinic during the study period. | 3,613 | Type 1 and type 2 |
| Price-Haywood et al., 2017 [33] | USA | Primary care | Patient 18 years or older with at least 2 primary care visits between July 2012 and December 2014, diagnosis with hypertension and/or diabetes. | 5,296 | Type 1 and type 2 |
| Reed et al., 2019 [34] | USA | Integrated healthcare system | All patients in the clinical chronic conditions registry for diabetes from January 2006-December 2007. | 165,47 7 | Unspecified |
| Shimada et al., 2016 [35] | USA | Integrated healthcare system | Patients with at least 2 outpatient records or 1 inpatient record with a diagnosis code for type 2 diabetes and registered to use MyHealthVet (MHV) portal. | 111,686 | Type 2 |
| Tenforde et al., 2012 [36] | USA | Primary care | Primary care patients aged 18-75, visiting primary care physician of the study location at least twice during study period. | 10,746 | Unspecified |
| 4. Cross-sectional studies | | | | | |
| Wade-Vuturo et al., 2013 [27] | USA | Primary care | Type 2 diabetes patients, prescribed antihyperglycemic medications through the Vanderbilt University Medical Center primary care clinic waiting rooms. | 54 | Type 2 |
