# Supplementary material for: Patient Portal Functionalities and Patient Outcomes Among Patients With Diabetes: Systematic Review
Source: J Med Internet Res. 2020 Sep 22;22(9):e18976. doi: 10.2196/18976 (PMC7539164; doi:10.2196/18976)
Supplement: Multimedia Appendix 5 [file jmir_v22i9e18976_app5.docx]

**Multimedia Appendix 5: [Risk of bias assessment results from applying the Cochrane Collaboration Risk of Bias Tool [21] for the randomised controlled trial by Grant et al. [26]]**

| Domain | Author's judgement | Support for Judgement |
| --- | --- | --- |
| Selection bias: Random sequence generation | Low | Probably done; The study "randomized at the practice level" and each practice was "randomly assigned" |
| Selection bias: Allocation concealment | High | Probably not done because it is not possible to conceal the intervention (patient portal) |
| Performance bias: Blinding (participants and personnel) | High | Probably not done because it is not possible to conceal the intervention (patient portal). The difference between the two portals was only the "content of the modules" as the portal used by both groups was the same. |
| Detection bias: Blinding (outcome assessment) | High | Probably not done as the study does not say it was a "double-blind" study |
| Attrition bias: Incomplete outcome data | Low | - |
| Reporting bias: Selective reporting | High | Data is not shown for blood pressure and LDL-Cholesterol control results |
| Other bias: Other sources of bias | Low | - |
