# Supplementary material for: Patient Portal Functionalities and Patient Outcomes Among Patients With Diabetes: Systematic Review
Source: J Med Internet Res. 2020 Sep 22;22(9):e18976. doi: 10.2196/18976 (PMC7539164; doi:10.2196/18976)
Supplement: Multimedia Appendix 6 [file jmir_v22i9e18976_app6.docx]

**Multimedia Appendix 6: [Risk of bias assessment results from applying the NHLBI - National Heart, Lung and Blood Institute quality assessment tool for observational cohort and cross-sectional studies]**

| Study | Overall rating |
| --- | --- |
| Chung et al., 2017 [28] | Fair |
| Devkota et al., 2016 [29] | Fair |
| Lau et al., 2014 [25] | Poor |
| Lyles et al., 2016 [30] | Good |
| McClellan et al., 2016 [31] | Fair |
| Petullo et al., 2016 [32] | Good |
| Price-Haywood & Luo, 2017 [33] | Fair |
| Reed et al., 2019 [34] | Good |
| Shimada et al., 2016 [35] | Good |
| Tenforde et al., 2012 [36] | Good |
| Wade-Vuturo et al., 2013 [27] | Poor |
